# Does the ^1^H-NMR plasma metabolome reflect the host-tumor interactions in human breast cancer?

**DOI:** 10.18632/oncotarget.18307

**Published:** 2017-05-30

**Authors:** Vincent Richard, Raphaël Conotte, David Mayne, Jean-Marie Colet

**Affiliations:** ^1^ Department of Medical Oncology, CHU Ambroise Paré, B-7000 Mons, Belgium; ^2^ Laboratory of Human Biology and Toxicology, Faculty of Medicine and Pharmacy, University of Mons, B-7000 Mons, Belgium; ^3^ UMHAP, Bioprofiling Unit, B-7000 Mons, Belgium; ^4^ Unité de Recherche Clinique, CHU Ambroise Paré, B-7000 Mons, Belgium

**Keywords:** breast cancer, metabolomics, ^1^H-NMR, plasma, profiling

## Abstract

Breast cancer (BC) is the most common diagnosed cancer and the leading cause of cancer death in women worldwide. There is an obvious need for a better understanding of BC biology. Alterations in the serum metabolome of BC patients have been identified but their clinical significance remains elusive. We evaluated by ^1^H-Nuclear Magnetic Resonance (^1^H-NMR) spectroscopy, filtered plasma metabolome of 50 early (EBC) and 15 metastatic BC (MBC) patients. Using Principal Component Analysis, Partial Least-Squares Discriminant Analysis and Hierarchical Clustering we show that plasma levels of glucose, lactate, pyruvate, alanine, leucine, isoleucine, glutamate, glutamine, valine, lysine, glycine, threonine, tyrosine, phenylalanine, acetate, acetoacetate, β-hydroxy-butyrate, urea, creatine and creatinine are modulated across patients clusters. In particular lactate levels are inversely correlated with the tumor size in the EBC cohort (Pearson correlation *r* = −0.309; *p* = 0.044). We suggest that, in BC patients, tumor cells could induce modulation of the whole patient's metabolism even at early stages. If confirmed in a lager study these observations could be of clinical importance.

## INTRODUCTION

Breast cancer (BC) is the most common diagnosed cancer and the leading cause of cancer death in women worldwide, accounting for 25% of all cancer cases and 15% of all cancer deaths among females. Mortality rate has declined over the last decades mainly due to advances in screening methods, leading to earlier diagnosis, and successful multidisciplinary treatments of the early diseases [1]. However, despite progresses in therapies and supportive cares of advanced diseases, the majority of relapsing patients die of the malignancy or its complications [2].

So there is an obvious need for a better understanding of BC biology.

Metabolomics, the global qualitative and quantitative evaluation of metabolites in a biological system, by NMR spectroscopy, mass spectrometry or combined techniques [3, 4] has emerged as a unique tool to investigate the modification of metabolites of cancer cells or in biofluids and tissues of cancer patients [5, 6].

Many studies have shown significant alterations in the plasma or serum of BC patients. However, because of the considerable diversity of these results, BC metabolomics remain exploratory [7–9]. Moreover, the origin of these systemic metabolic modifications remains a subject of debates. At first glance they could be considered as direct leakages of cancer cells metabolites but recent reports suggest that host response to cancer is also important even at early stages [10]. The serum composition, as far as the endogenous metabolites are considered, could be remodeled due to such host-tumor interactions [7, 8, 10].

We report here a ^1^H-NMR-based metabonomic study describing metabolic plasma modifications across EBC and MBC patients cohorts and tentatively propose some biological explanations opening new putative perspectives for BC metabonomics evaluation and the management of patients.

## RESULTS

### Population and clinico-pathological parameters

Fifty EBC and fifteen MBC patients were included in this study. Two patients with non-invasive histology were finally excluded from metabolomics analysis.

### EBC plasma samples analyses

A first PCA analysis blindly performed on all samples identified five outliers: one patient had very high lactate plasma level probably due to inadequate blood collection or manipulation, two presented high ethanol levels and two patients showed high glucose levels due to non insulin dependent diabetes. They were all excluded from further analyses.

**Figure 1:**
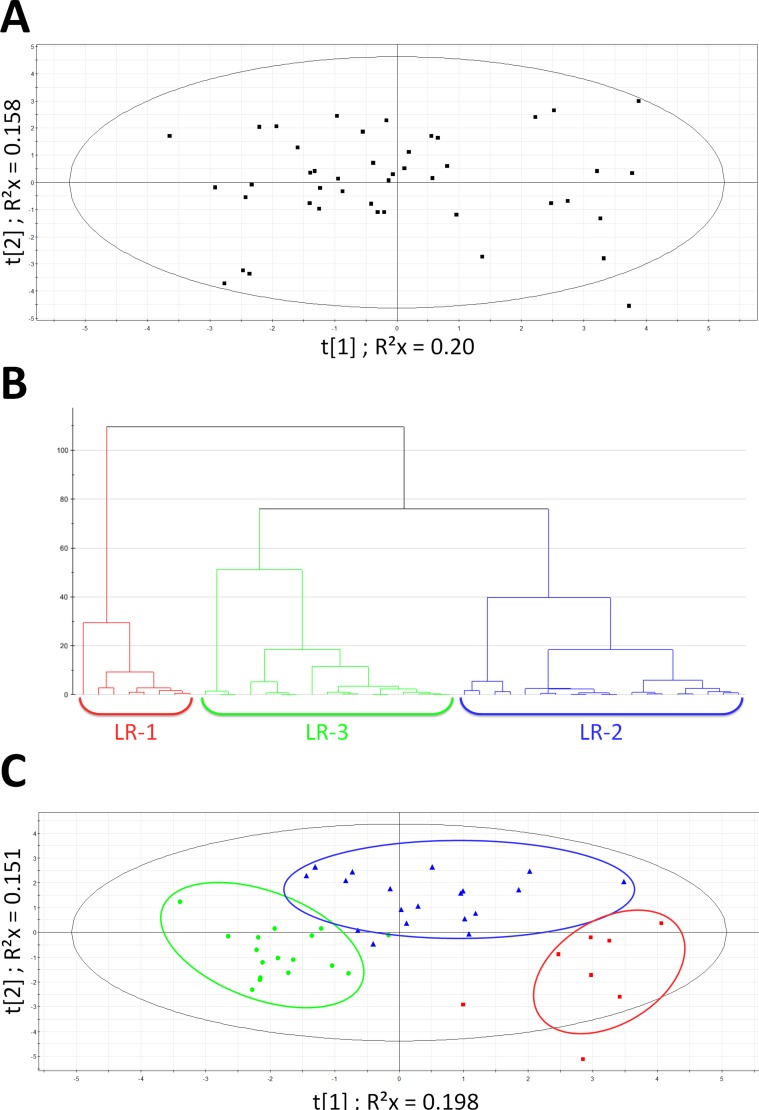
Multivariate statistical analysis on EBC population (**A**) Scores scatter plot obtained after PCA on EBC population. Model parameters: R^2^cum = 0.737; Hotelling T2 = 0.95. Eigth proposed principal components (**B**) Hierarchical clustering on EBC population: 3 patient's subgroups emerged: LR-1 (red), LR-2 (blue) and LR-3 (green). The distance is calculated with Ward and sorted by size. (**C**) PLS-DA on EBC population - clusters defined by HC : LR-1(red square); LR-2 (blue triangle); LR-3 (green dot). Model parameters: R^2^Xcum = 0.35; R^2^ Ycum = 0.649; Q^2^cum = 0.519 Hotelling T2 = 0.95; Two proposed principal components.

As shown in Figure 1, PCA (A), hierarchical analyses (B) and PLS-DA (C) next allowed to significantly split up the whole EBC population into 3 clusters named LR-1, LR-2 and LR-3 (CV-ANOVA *p* < 0.001; permutations parameters: R^2^ = [0.0, 0.242]; Q^2^ = [0.0, −0.233]). Table 1 outlines the main characteristics of both patients and tumors in the whole EBC population as well as in the three clusters identified by the multivariate analysis. We did not find any significant clinical differences between these clusters, except for the younger age in LR-2 (ANOVA *p* = 0.014). Although not significant (Kruskal-Wallis test *p* = 0.131), a trend towards a smaller mean tumor size in cluster LR-1 as compared to LR-3 (See Table 1 for data) was observed. Identified metabolites with VIP values ≥ 1 were: lactate, glucose, pyruvate, glutamine, glutamate, alanine, valine, leucine, glycine, creatine, creatinine, urea, acetate, acetoacetate and β-hydroxyburyrate.

**Table 1:** Characteristics of patients and tumors of whole EBC population and of multivariate data analysis defined clusters

GROUPS	TOTAL POPULATION	LR-1	LR-2	LR-3
**Total number of patients**:	43	8	19	16
**Mean age (years)**:	56 (29–82)	61.5* (48–76)	50.2 (29–65)	59* (44–82)
**BMI (Kg /m^2^)**	29 (14 .8–37.8)	29.2 (19.8–37.8)	25.9 (14.8–31.6)	25.4 (20.4–31.5)
**Tumor histological type**:	Number (%)			
Invasive carcinoma NST	37 (86)	8 (100)	17 (89)	12 (75)
Lobular invasive carcinoma	6 (14)	0	2 (11)	4 (25)
**TUMOR STADIFICATION**	Number (%)			
T1	23 (53.5)	7 (87.5)	9 (47.3)	7 (43.7)
T2	10 (23.2)	1 (12.5)	4 (21.5)	5 (31.2)
T3–T4	10 (23.2)	0	6 (31.5)	4 (25.0)
Positive node(s)	8 (18.6)	2 (25)	4 (15.7)	3 (18.75)
**Median Tumor size (mm)**	27.1	13	15	21
**TUMOR INTRINSIC SUBTYPES**	Number (%)			
Luminal A	17 (39.5)	5 (62.5)	4 (21)	8 (50)
Luminal B	17 (39.5)	2(25)	10 (52.6)	5 (31.2)
Luminal B Neu amplified	2 (4.6)	1 (12,5)	1 (5)	0 (0)
Neu amplified	2 (4.6)	0	1 (5)	1 (6.2)
Triple Receptor Negative	5 (11.6)	0	3 (15.7)	2 (12.5)

Statistical Analyses: *significant level (*p* < 0.05) compared to LR-2.

ANOVA was used for BMI and age; if ANOVA test significant, Pairwise Multiple Comparison Procedures (Tukey test) were used.

Kruskal-Wallis on Ranks were used for tumor size.

Fischer Test was used for tumor staging and tumor intrinsic subtypes.

Table 2 shows the VIP and AUC values of the discriminant metabolites. Lactate, pyruvate, alanine levels were significantly higher and acetate, glucose and glutamine levels were significantly lower in cluster LR-1 compared to clusters LR-2 and LR-3. After Bonferroni adjustment, the differences observed in lactate, pyruvate and glutamine levels remained statistically significant (α Bonferroni = 0.0033).

**Table 2:** Levels (AUC) of discriminant metabolites in EBC clusters LR-1, LR-2 and LR-3

METABOLITES	δ^1^H (ppm)^a^	VIP value	AUC (LR-1)^b^	AUC (LR-2)^b^	AUC (LR-3)^b^	*p* value^c^
**CARBOHYDRATES**						
LACTATE	1.32	13.76	11.31 (10.8–11.89)	7.91* (6.72–8.54)	5.87*^$^ (5.51–6.33)	**< 0 .001**
PYRUVATE	2.36	2.02	0.50 (0.45–0.56)	0.36* (0.3–0 .41)	0.34* (0.31–0.36)	**< 0.001**
GLUCOSE	3.76	2.55	2.90 (2.7–3.09)	3.23 (3.03–3.29)	2.98 (2.75–3.13)	0.008
**CETONIC BODIES**						
ACETATE	1.92	1.65	0.19 (0.17–0.22)	0.18 (O.13–0.19)	0.21 (0.18–0.24)	0.025
ACETO-ACETATE	2.28	1.50	0.13 (011–0.16)	0.12 (0.09–0.21)	0.16 (0.12–0.26)	0.20
3-HX-BUTYRATE	4.16	1.48	0.18 (0.14–0.32)	0.20 (015–0.35)	0.22 (0.11–0.67)	0.92
**AMINO ACIDS**						
ALANINE	1.46	0.98	1.95 (1.78–2 .29)	1.56 (1.44–1.87)	1.60 (1.55–2.05)	0.026
GLUTAMINE	2.42	1.20	1.50 (1.44–1.53)	1.48 (1.31–1.58)	1.68^$^ (1.59–1.78)	0.003
GLUTAMATE	2.00	1.12	0.43 (0.35–0.52)	0.32 (0.27–0.39)	0.48 (0.29–0.65)	0.15
LEUCINE	0.96	1.33	0.86 (0.77–1.09)	0.93 (0.78–1.04)	1.00 (0.89–1.07)	0.43
VALINE	0.98	0.95	0.93 (0.77–1.04)	0.87 (0.74–0.99)	0.89 (0.82–1.05)	0.75
GLYCINE	3.56	1.28	0.66 (0.58–0.86)	0.66 (0.48–0.80)	0.71 (0.49–0.95)	0.48
**UREA METABOLITES**						
UREA	5.79	1.05	0.25 (0.19–0.47)	0.33 (026–0.66)	0.49 (0.32–0.96)	0.30
CREATININE	4.06	1.6	0.21 (0.19–0.23)	0.22 (0.19–0.25)	0.26 (0.23–0.3)	0.07
CREATINE	3.94	1.41	0.24 (0.21–0.29)	0.23 (0.16–0.28)	0.29 (0.23–0.33)	0.18

A: Chemical shift

B: Area Under the Curve (AUC): median (25%–75%).

C: Kruskal-Wallis One Way Analysis of Variance on Ranks and if significant Pairwise Multiple Comparison Procedures (Tukey test): After Bonferroni correction significant *p* level value < 0.003(*: significant compared to LR-1; ^$^: significant compared to LR-2).

**Figure 2:**
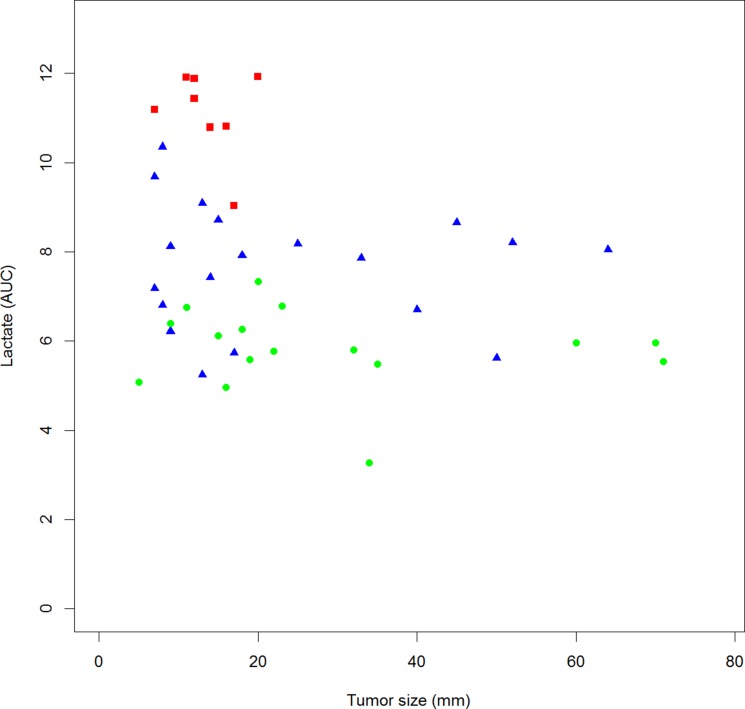
Correlation between lactate plasma level and tumor size in EBC patients Square plot, LR-1(red square); LR-2 (blue triangle); LR-3 (green dot) Pearson Correlation *r* = −0.309; *p* = 0.044.

We show a significant inverse correlation between lactate levels and tumor size (Figure 2; Pearson correlation *r* = −0.309; *p* = 0.044). This correlation does not reach significance for pyruvate, alanine, glucose, acetate and glutamine levels (data not shown). Correlations between metabolites levels are shown in Table 3. Interestingly, there is a significant positive correlation between lactate, pyruvate and alanine levels as well as a significant negative correlation between alanine and glucose levels. Correlation between lactate and glucose levels is negative but not significant.

**Table 3:** Correlations between AUC metabolites in the EBC population

	Acetate 1.92 ppm	Alanine 1.48 ppm	Glucose 5.24 ppm	Glutamine 2.46 ppm	Lactate 1.34 ppm	Pyruvate 2.38 ppm
**Acetate 1.92 ppm**	---					
**Alanine 1.48 ppm**	0.2270	---				
**Glucose 5.24 ppm**	−0.4793*	−0.4015*	---			
**Glutamine2.46 ppm**	0.2822*	0.1513	−0.2989*	---		
**Lactate 1.34 ppm**	−0.2212	0.3206*	−0.1962	−0.3395*	---	
**Pyruvate 2.38 ppm**	−0.1296	0.3146*	−0.3114*	−0.1729	0.6821*	---

In green: positive correlation and in red: negative correlation.

Pairwise two-side test: *p value < 0.05.

### MBC plasma samples analyses

PCA and HC analyses tended to separate the whole MBC population into two main clusters (MT-1 and MT-2) and a cluster of 2 patients (MT-3) (data not shown).

Table 4 outlines the main patients and tumors characteristics of the whole MBC population and of the HC defined clusters. Due to its small size, MT-3 cluster did not allow robust PCA and statistical analyses. It was eventually excluded.

**Table 4:** Clinico-pathological characteristics of the mbc cohort and of the clusters identified by multivariate data analysis

GROUPS	TOTAL POPULATION (range)	MT-1 (range)	MT-2 (range)	MT-3 (range)
**Total number of patients**:	15	5	8	2
**Mean age (years)**:	62 (44–83)	66 (58–75)	61 (44–83)	57 (53–62)
**BMI (Kg /m^2^)**	24.8 (19.9–33.6)	26.7 (21.3–33.9)	24.4 (20–33.6)	NA
**Tumor histological type**	Number (%)			
Invasive carcinoma NST	13 (87)	3 (60)	8 (100)	2 (100)
Lobular invasive carcinoma	2 (13)	2 (40)	0	0
**TUMOR INTRINSIC SUBTYPES**	Number (%)			
Luminal A /B	9 (60)	3 (60)	4 (50)	2 (100)
Neu Amplified	2 (13)	0	2 (25)	0
Triple Receptor Negative	4 (27)	2 (40)	2 (25)	0
**METASTATIC SITES**	Number (%)			
Bone only	3 (20)	2 (40)	1 (12.5)	1 (50)
Liver	6 (40)	2 (40)	4 (50)	0
More than 1 site	7 (47)	2 (40)	5 (62,5)	1 (50)
**First line metastatic therapy**	10 (67)	3 (60)	6 (75)	1 (50)
**Mean overall survival (OS) (months)**	17.6 (2–45)	23.6	14.3	15

Welch two sample *t*-test was used for age, BMI and OS.

Fisher test was used for “more than one site” parameter, significant *p* level< 0.05.

**Figure 3:**
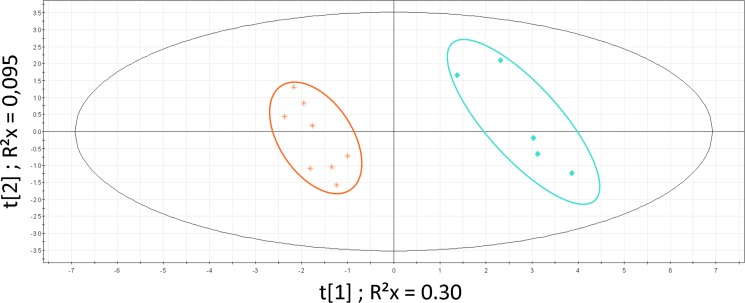
PLS-DA on MBC population Patients clusters defined by HC: MT-1 (blue diamond) and MT-2 (orange star). Model parameters: R^2^Xcum = 0.398; R^2^Ycum = 0.976; Q^2^cum = 0.869; Hotelling T2 = 0.95, Two proposed principal components.

PLS-DA analysis restricted to the 2 main clusters (MT-1 and MT-2) was significant (Figure 3; CV-ANOVA *p* = 0.001; permutations parameters R^2^ = [0.0, 0.832]; Q^2^ = [0.0, −0.252]). We did not find any significant clinical difference between these clusters. Although not significant, patients in the cluster MT-2 presented a shorter overall survival and a more extended disease (more than one metastatic site) than patients from cluster MT-1 (14.3 months vs 23.6 months; *t*-test *p* = 0.3). Identified metabolites with VIP values >1 were: glucose, lactate, alanine, leucine, isoleucine, glutamate, glutamine, valine, lysine, tyrosine, phenylalanine, threonine, β-hydroxy-butyrate, acetate, acetoacetate, urea, creatine and creatinine.

According to the AUC, lactate and glucose levels were significantly higher and glutamate, valine, lysine, tyrosine, phenylalanine, creatinine, acetate and acetoacetate were significantly lower in cluster MT-2 as compared to cluster MT-1. After Bonferroni adjustment (α Bonferroni = 0.0027) only the differences observed in lysine, tyrosine, and phenylalaline levels remained statistically significant. Table 5 shows the VIP AUC values of the discriminant metabolites.

**Table 5:** Levels (AUC) of discriminant metabolites in MBC clusters MT-1 and MT-2

METABOLITES	δ^1^H (ppm)^a^	VIP value	AUC (MT-1)^b^	AUC (MT-2)^b^	*P* value^c^
**CARBOHYDRATES**					
LACTATE	1.32	6.94	5.40 (4.18–5.74)	6.39 (6.02–8.78)	0.006
GLUCOSE	3.46	4.64	2.89 (2.76–3.02)	3.35 (3.11–3.64)	0.003
**CETONIC BODIES**					
ACETATE	1.92	2.38	0.21 (0.18–0.25)	0.16 (0.14–0.17)	0.011
ACETO-ACETATE	2.28	1.50	0.18 (0.15–0.55)	0.10 (0.06–0.16)	0.045
Β-HYDROXYBUTYRATE	1.20	1.21	0.16 (0.12–1.03)	0 .16 (0.08–0.24)	0.524
**AMINO ACIDS**					
GLUTAMATE	2.14	1.84	0.51 (0.45–0.58)	0.38 (0.34–0.44)	0.011
GLUTAMINE	2.46	1.84	1.54 (1.34–1.61)	1.23 (1.05–1.53)	0.09
VALINE	0 .98	2.79	0.96 (0.88–1.21)	0.74 (0.51–0.86)	0.011
ALANINE	1.46	1.27	1.54 (1.37–1.93)	1.43 (1.27–1.73)	0.28
LEUCINE	0.96	1.82	0.89 (0.84–1.32)	0.74 (0.67–0.83)	0.065
ISOLEUCINE	0.94	2.06	0.25 (0.23–0.39)	0.19 (0.16–0.21)	0.018
LYSINE*	1.90	2.16	0.94 (0.85–1.11)	0.69 (0.53–0.72)	**0.001**
TYROSINE*	7.19	1.56	0.23 (0.19–0.33)	0.16 (0.11–0.17)	**0.001**
PHENYLALANINE*	7.33	1.13	0.18 (0.17–0.19)	0.12 (0.12–0.14)	**0.001**
THREONINE	4.24	1.11	0.19 (0.15–0.22)	0.09 (0.06–0.12)	0.06
**UREA METABOLITES**					
CREATININE	4.06	1.28	0.53 (0.42–0.58)	0.39 (0.25–0.47)	0.045
CREATINE	3.92	2.33	0.37 (0.17–0.45)	0.23 (0.12–0.36)	0.28
UREA	5.81	2.06	1.01 (0.68–1.72)	0.62 (0.29–0.92)	0.065

a: chemical shift

b: Area Under the Curve: median (25–75%)

c: Wilcoxon test: Statistically Significant (*p* < 0.05). After Bonferroni correction, significant *p* value = 0.0027

*Significant *p* level.

### Levels (AUC) of the discriminant metabolites across the whole population, EBC and MBC patients

The same data analyses as described above were applied to the whole study population (EBC and MBC patients). We obtained significant separation (CV-ANOVA *p* < 0.001) between groups of patients although some overlaps were noticed. We identified the same set of discriminant metabolites as already reported in EBC and MBC patients (data not shown; PLS-DA model parameters: R^2^ Xcum = 0.351;R^2^ Ycum = 0.353; Q^2^ cum = 0.224; two proposed components).

**Figure 4:**
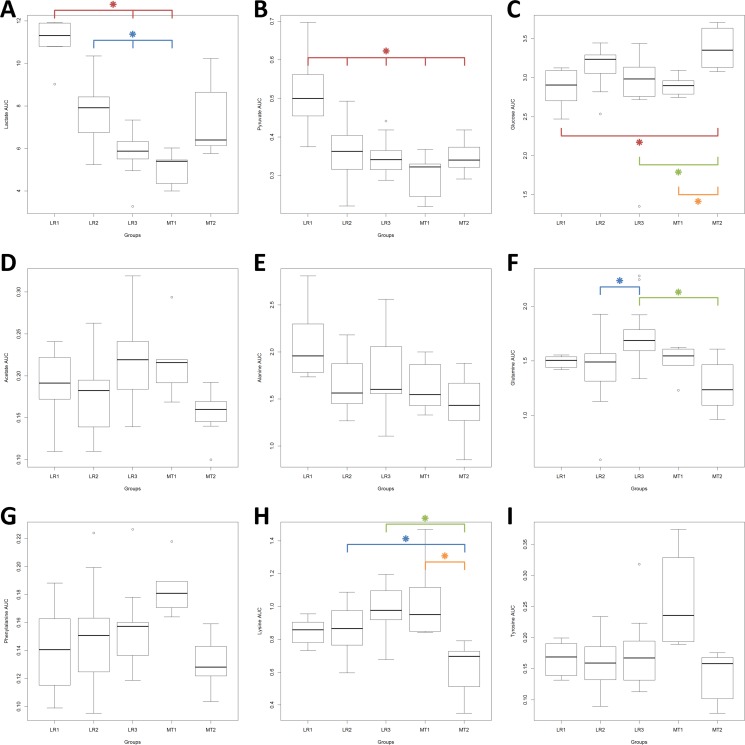
Levels (AUC) of the discriminant metabolites across the whole population, EBC and MBC patients Box plots: horizontal line within the box = mean; bottom and top lines of the box = 25th and 75th percentiles, respectively. Kruskal-Wallis One Way Analysis of Variance on Ranks, *p* significant < 0.05. After Bonferroni correction, significant *p* value = 0.0045. Pairwise Multiple Comparison Procedures (Dun's Method), *p* significant < 0.05. Red star : compared to LR-1; blue star : compared to LR-2; green star : compared to LR-3: orange star: compared to MT-1.

In Figure 4, the levels (AUC) of nine of these discriminant metabolites (lactate, pyruvate, alanine, glucose, glutamine, acetate, phenylalanine, lysine and tyrosine) showed significant differences across the whole study population (Kruskal-Wallis *p* < 0.05). Differences of levels for valine and urea nearly reached significant scores (Kruskal-Wallis *p* = 0.052 and 0.055, respectively – data not shown) After Bonferroni adjustment (α Bonferroni = 0.0045) only the differences observed in lactate, pyruvate, glucose glutamine and lysine levels remained statistically significant.

## DISCUSSION

In the field of BC, several ^1^H-NMR based metabonomic studies compared plasma or serum metabolites profiles between EBC and MBC patients or dissected the EBC or MBC profiles themselves [9, 14] A few compared EBC patients to healthy controls [15, 16] and one study recently reported a comparative evaluation between BC and other cancer types [17]. From these results, the concept of serum or plasma metabolonomic signatures of BC has emerged with the hope of improving the prediction and early detection of relapses. However, due to the diversity of the reported metabolomics signatures of BC, the approach still remains exploratory [7, 8]. Correlations between ^1^H-NMR serum or plasma metabolomics signatures and either the primary tumor characteristics in EBC or the severity of disease in MBC remain elusive [9, 11], except for the association between lipoprotein subfractions and EBC tumor characteristics in one report [18]. Some authors suggest that the metabolomics profiles could be the result of the presence of BC cells themselves and the host-tumor interactions [8, 10].

With this last question in mind we evaluated by ^1^H-NMR spectroscopy, the plasma metabolome of early and metastatic BC patients. In accordance with the literature, our results show that the plasma levels of a set of metabolites are modulated across different sub-groups of EBC and MBC patients [7, 8].

Our results highlight the heterogeneity of the plasma metabolites inside the EBC and MBC populations and the differences between the two clinical situations. The absence of matching healthy control group strictly limits our conclusions to the pathologic situation but we think that the evolution of the metabonomic profile inside the EBC group and the results published by others could authorize some speculations about the tumor impacts on the systemic metabolism.

In the following discussion, we will propose a putative coherent metabolic model to explain these systemic metabolic changes. Across the EBC subgroups, we show statistically significant modulations of several metabolites: lactate, pyruvate, glutamine, glucose, alanine and acetate; these three last ones however not remaining significant after Bonferroni adjustment. We also find significant correlations between levels of some of them. Lactate, pyruvate and alanine are positively correlated; pyruvate and alanine are negatively correlated with glucose; glutamine is negatively correlated with glucose and lactate. Moreover, we observe a weak but significant inverse correlation between lactate levels and the tumor size, smaller tumors being associated with higher plasma lactate levels. Correlations between tumor size and the other metabolites are not significant. To our knowledge, these observations have never been reported in the metabonomics breast cancer literature.

A first explanation could be that this weak but significant correlation could be linked to the small LR-1 patients subgroups exhibiting higher BMI and higher mean age, these clinical parameters influencing the metabolites levels. Actually Psychogios et al. reported important variations of lactate serum levels determined by metabolomic studies in healthy populations, due to many parameters such as BMI and age [4]. However, the BMI differences are not statistically different between our EBC sub-groups and we did not find any significant correlation between BMI and the metabolites levels. Recently published metabolomics studies reported somewhat contradictory results on BMI and human blood metabolite profiles [19, 20].

A second hypothesis to explain these observations could be that cancer cells and their microenvironment consume lactate leading to its levels drop with growing tumor mass. Several facts do not support such a speculation: cancer cells themselves produce and excrete large amounts of lactate in their stroma where this metabolite plays subtle and complex roles favoring tumor progression [21, 22]; Kallinowski F. et al. showed that in human breast cancer xenografts in nude rats, lactate is released from tumors [23]; Kennedy KM. et al. found that, *in vitro*, lactate can be metabolized by some but not all types of BC cells [24]. Moreover this hypothesis would not explain the modulations observed for other metabolites and it is not accurate to transpose local tumor metabolism to the whole human body [25]. So, we are not convinced that the drop in lactate levels and other associated systemic metabolic alterations are directly linked to the tumor metabolism.

The observation that the modifications of lactate, pyruvate and alanine levels inside the EBC groups are positively correlated leads us to formulate a third hypothesis. We would suggest that the drop in the lactate plasma levels observed in EBC, associated with the modifications of pyruvate and alanine, is a clue of the activation of the lactate cycle (Cori cycle) and liver neo-glycogenesis. Lactate, released by muscles, and breast tumor in this case, is transported to the liver where it regenerates pyruvate and glucose avalaible for systemic metabolism. Alanine produced by transamination of pyruvate in muscles is transported to the liver where it enters neo-glycogenesis after inverse transamination to pyruvate. Pyruvate itself can be metabolized to lactate or be transported to the liver, entering also neo-glycogenesis. So the metabolism of these three metabolites and the increased activity of Cori cycle concur to a net increase of blood glucose, available to cancer cells and their stroma, and a decrease of lactate and glucogenic amino acid (AA) such as alanine. This phenomenon has been proposed many years ago to explain the disparity observed in lactate blood levels in different types of cancers [26–28]. Levin et al. suggested that elevated glucose level in blood cancer patients could be the result of increased gluconeogenic flux from lactate and glucogenic amino acids [29]. Altered glucose metabolism in metastatic carcinoma has been known since as long as 1975 [30]. It has been suggested more recently that activation of so-called metabolic futile cycles, such as Cori cycle, could contribute to cancer cachexia [31]. DeBernardinis et al. suggested cancer-muscle relation through glutamine and Luo Y et al. published data supporting this hypothesis at least *in vitro* [32, 33]. However, these publications were in advanced cancer situation.

Our data suggest that cancer-related neo-glycogenesis is active at early stages and increases with progressive malignant disease as suggested by the metabolic evolution across EBC. We acknowledge that the lack of healthy control group weakens our hypothesis. In a preliminary small study using the same NMR techniques, on unfiltered residual serum samples, we showed that some, but non significant, separation between EBC and healthy women was mainly due to lipids fractions, glucose, alanine, lactate, β-hydroxy-butyrate and acetate (Oral presentation in ESMRMB Congress, Lisbon 2012). Although immature, these data are in line with other publications. We would like to pinpoint the MS based metabolomic study of Shen et al. They observed that plasma alanine levels were lowered in EBC patients compared to healthy controls and especially in patients bearing the aggressive TRN tumors. No information are available on tumor size. [34]. Interestingly, in another study evaluating serum ^1^H-NMR metabolomic profile of EBC and responses to chemotherapy, the combination of lactate, alanine and glucose levels were significant in multivariate analysis to predict tumor response [35]. Gu H. et al. identified lactate, glucose and alanine among other metabolites discriminating serum samples collected from BC patients and healthy controls [16].

Coming back to our data, we observed that AAs are heavily involved across the whole study population: alanine, leucine, isoleucine, glutamate, glutamine, valine, lysine, glycine, threonine, tyrosine, and phenylalanine. All AAs identified but the cetogenic leucine and lysine, are glucogenic, which is coherent with the neoglucogenic-cancer induced hypothesis. Interestingly glutamine levels are rising significantly across the EBC subgroups then falling in MBC cohorts. It has been proposed that this AA is exported from the muscle to the tumor to participate to its protein synthesis or as a metabolic fuel for the Krebs cycle [31].

Comparing the serum levels of 15 AAs as measured by high performance liquid chromatography on serum EBC patients to healthy volunteers, Poschke et al. published significant increased levels of glutamine, glutamate, serine, alanine, valine, leucine. There was no significant correlation with tumor stage but the basal like tumors patients had the highest AAs serum levels [36]. Huang S. et al., using mass spectrometry, identified discriminant blood metabolites in BC patients at all-stages and measured their average log-fold changes versus control samples. From these data, they constructed models to separate early stage BC from all-stage BC. They observed that glutamine levels were higher and alanine levels lower during early tumorigenesis [37]. Other studies reported contradictory results but showed a clear effect of BC on patient AAs metabolism [37, 38].

We suggest that, even in EBC, the tumor could induce some protein catabolism with the release of some neoglucogneic AAs. However, sarcopenia was not evaluated in our study and there was no significant correlation with the BMI, but it is known that whole-body protein turn-over rates are increased in cancer patients prior to clinical cachexia signs [39]. In MBC patients, we observed a drop in all the already identified AAS but glycine, with the worsening of the disease, potentially reflecting the exhausted MBC patient metabolism.

Our data could be interpreted as signs of an activation of neoglucogenesis in BC patients, using lactate and neoglucogenic AAs, increased proteins muscle catabolism with release of AAs. These systemic metabolism modifications seem to occur early in the disease. Liu L. et al. showed metabolic shifts induced by human lung cancer cells line in tumor-bearing mice [40]. This is in line with the evolution of the literature on the shifts in metabolism in cancer patients. A shift in energy metabolism in cancer cells was first described by Otto Warburg and was termed as « aerobic glycolysis » [41]. Progressively, many other evidences have accumulated leading to the larger and more complex concept of «°reprogramming energy metabolism°», recently qualified as an emerging hallmark of cancer [42]. Since many years it was also recognized that the presence of a cancerous tumor could induce global and systemic alterations in the patient's metabolism. However the mechanisms allowing a few cells to influence the whole metabolism were largely unknown [28, 29, 43, 44]. Recently it was even suggested that solid tumor behave as systemic metabolic dictators and the molecular basis of cancer cachexia have been outlined [45, 46]. It is proposed that cancer interacts with muscle, liver, and adipose tissue metabolisms to maintain its growth at the expense of the patient homeostasis. Inflammatory state, induced by and against the tumor could be the driving force leading to these metabolic modifications and several cytokines seem to be involved [45, 31, 47]. We recently published data showing the involvement of the Macrophage Migration Inhibitory Factor (MIF) in BC. MIF serum levels were significantly increased in EBC [48, 49]. Interestingly, this pleiotropic and proinflammatory cytokine is also involved in glucose metabolism and in the development of diabetes, insulin resistance and obesity which is a known risk factor for BC and recurrence of this disease [50–53].

The main pitfalls of our study are the relatively small numbers of patients, its transversal design and the data acquisition by 1D ^1^H-NMR analytical technique alone. Main drawbacks of 1D-NMR spectroscopy approach are its sometimes-difficult metabolites identification and semi-quantitative only evaluation. It so could be of interest to validate our data by a MS-based technique, considered more sensitive, more specific and providing more accurate quantification. The advantage of combining the two techniques has been reviewed recently [54, 55].

Our goal was to dissect the systemic metabolites modifications occurring at various stage of BC disease, and try to propose biological and physiological explanations, not to establish a prognostic or diagnostic signature. We think that our findings deserve further investigations on a larger scale because, if confirmed, they could contribute to innovative ways of BC management. It is of interest to note that one recent publication developed a model based on more than 500 preoperatively sera EBC ^1^H-NMR metabolomics profiles that seems to be prognostic for recurrence. Higher serum levels of lactate, valine, leucine, isoleucine, phenylalanine tyrosine, histidine, glutamate, glycine, among others, characterized the higher risk metabolomics profile. The authors suggest that the metabolomic signal is the result of host state and tumor cells [10]. In MBC patients, monitoring metabolomics changes along the treatments could help to establish a signature of therapeutic response. To our knowledge there is no data published on the subject and the management of cancer cachexia remains a problem [56].

From a therapeutic research point of view, we suggest that drugs with inhibitory effects on cytokines involved in the metabolic processes described could be of interest. For example, metformin, a well-known oral antidiabetic drug has recently received great attention as a potential BC therapy. Mechanisms of action are not yet established [57] but is noteworthy to remember that metformin can suppress plasma MIF concentrations in obese patients [58]. A phase II study is ongoing to explore the interest of this drug for reduction of obesity-associated BC risk, using plasma metabolomic profiles evaluation [59].

In conclusion, we suggest that BC could induce systemic metabolic modifications in patients even at early non-metastatic stages and worsening with the progression of the disease. Cytokines produced by the tumor and its microenvironment could be involved in the process. If confirmed by a larger study, this finding could be of research and clinical interest.

## MATERIALS AND METHODS

### Patient populations

This observational study was conducted at the Ambroise Paré Hospital (Mons, Belgium). Between July 2012 and March 2014, we prospectively collected blood samples from early (EBC) and metastatic BC (MBC) women.

All enrolled patients were women 18 years old or older with proven histology of invasive BC. They accepted to sign informed consent before inclusion. All early stage patients were newly diagnosed and free of any treatment prior to serum collection. Tumors were evaluated by bilateral mammography, echography and echography guided biopsy. Breast Magnetic Resonance Imaging was systematically performed, leading to second look echography and biopsy in case of additional suspect lesion(s). The metastatic population could have received one or more BC metastatic treatments, had to be progressive and blood collection was performed before any new BC treatment administration. In both cohorts, cancer staging work-up was performed following international guidelines [60, 2]. Tumors were classified according to the World Health Organization 2012 [61] and the American Joint Committee on Cancer Cancer Staging Manuel (TNM), Seventh Edition. Estrogen receptor (ER), progesterone receptor (PR) status, Ki-67 labeling index, expression and/or HER2 gene amplification were routinely performed following international recommendations [62–64]. Positivity for ER and PR was defined as an Allred score equal or superior to 3 [62]. HER 2 was considered positive according to American Society of Clinical Oncology (ASCO) guideline [65]. Breast cancer intrinsic subtypes were defined according to the St Galen 2015 Consensus [60]. According to local lab values, the distinction between luminal A and luminal B was based on a 15% Ki-67 cut-off [66]. All subjects with insulin-dependent diabetes, cardiac or renal failure, active infections, chronic inflammatory diseases, chronic systemic corticoids medication, history of non-BC invasive malignancies were excluded.

Investigation has been conducted in accordance with the ethical standards and according to the Declaration of Helsinki and to national and international guidelines and has been accepted by the members of the institutional review board: The Ethics Committee of Ambroise Paré Hospital (Mons, Belgium) approved this study on May 2012 according to the international and Belgian laws.

### Plasma samples preparation

Venous blood sample, from fasting patients, was taken before any treatment, on 5 ml EDTA vacutainer tube, centrifuged at 3,000 × g for 15 min at 4°C within 2 hours. Supernatant (plasma) was then split up into 2 tubes and immediately stored frozen at −80°C.

### ^1^H-NMR samples preparation and spectroscopy

In order to prepare samples for metabolomic analysis by ^1^H-NMR spectroscopy, 500 μL of plasma were filtered using 3kDa Amicon^®^ Ultra-0.5 ml Centrifugal Filter Devices (Millipore) at 14,000 x g during 30 minutes. The filter was initially prewashed four times with distilled water to remove any traces of preservatives. Filtered proteins were rinsed using an additional 150 μL of D_2_O. Filtrates samples were then transferred to tubes with 100 μL of phosphate buffer (NaH_2_PO_4_ 0.04M, Na_2_HPO_4_ 0.2 M, pH 7.4) containing 3.5mM 3-(trimethylsilyl) -propionic-2,2,3,3-d4 acid (TSP) and prepared in 100% D_2_O.

600 μL of filtered plasma sample were transferred into 5 mm NMR tube and analyzed on a Bruker Avance 500 spectrometer (11.8 T) at 500 MHz for proton observation within a 5 mm BBI 1H/D-probe. One-dimensional spectrum was acquired at 297^°^K using a NOESYPRESAT-1d pulse sequence.

128 free induction decays (FID) with 54,832 data points per FID were collected for each sample using a spectral width of 10,330.578 Hz, an acquisition time of 2.65 sec, and a pulse recycle delay of 3 sec.

After proton 1D-NMR acquisition, FID signal was imported into MestReNova 10.0 Software (Mestrelab Research, Santiago de Compostela, Spain) for Fourier transformation and a line broadening of 0.3 Hz was applied. Then the spectra were automatically phase-and baseline-corrected and calibrated against TSP. The resonance of the methyl groups in TSP were arbitrarily placed at 0.00 ppm.

Spectral region from 0.08 to 10.00 ppm was automatically reduced to 496 integrated regions (buckets) of 0.02 ppm width each. The regions from 4.50 to 5.20 ppm containing residual water signal were removed, as well as two regions containing EDTA resonances from 3.20 to 3.22 ppm and from 3.58 to 3.62 ppm and three regions containing contamination from sample storage or venepuncture containers (from 2.54 to 2.56 ppm, 2.7 ppm, and from 3.06 to 3.18 ppm).

Each integrated region was normalized to the total spectrum area.

### Multivariate statistical analysis

The integrated reduced data were imported into SIMCA-P+12 (Umetrics, Umea, Sweden) for Principal Component Analysis (PCA), Partial Least-Squares Discriminant Analysis (PLS-DA) and Hierarchical Clustering Analysis (HCA, dendrogram), as described previously [67, 68]. All data were mean-centred and Pareto scaled [69].

In a first approach, we performed unsupervised analyses by PCA and HCA on data from each group of patients considered separately. PCA was used to evaluate the degree of homogeneity, identified outliers and outlined sub-groups. HCA allowed us to isolate clusters of patterns and to build a dendogram. The distance was calculated with Ward and sorted by size. To this respect, it has to be stressed out that no objective method is available yet to mathematically validate the partitioning of the data and, consequently, the clusters are intuitively defined. This is why the clustering models were then analysed by PLS-DA (with Q^2^ cum> 0.4), allowing their validation (CV–ANOVA and permutations test) [70]. The variables of importance in the projection (VIP) lists were then established. Using a cut-off value of VIP ≥ 1, discriminant metabolites were pinpointed for each cluster. In a second step, EBC and MBC data were mixed together and analysed using the above-described process.

### Identification and quantification of metabolites

**Figure 5:**
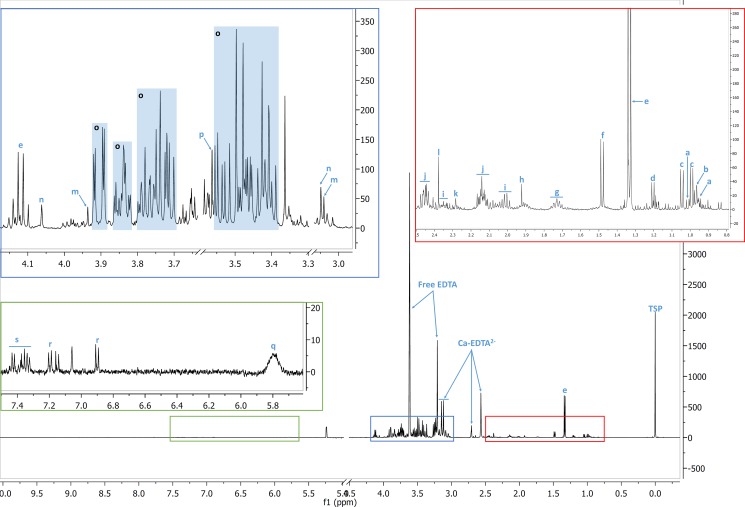
Identification of the metabolites A typical ^1^H-NMR spectrum of EBC patient filtered plasma. a: isoleucine; b: leucine; c: valine; d: β-hydroxybutyrate; e: lactate; f: alanine; g: lysine; h: acetate; i: glutamate; j: glutamine; k: acetoacetate; l: pyruvate; m: creatine; n: creatinine; o: glucose; p: glycine; q: urea; r: tyrosine; s: phenylalanine. TSP: 3-(trimethylsilyl) -propionic-2, 2,3,3-d4 acid.

**Figure 6:**
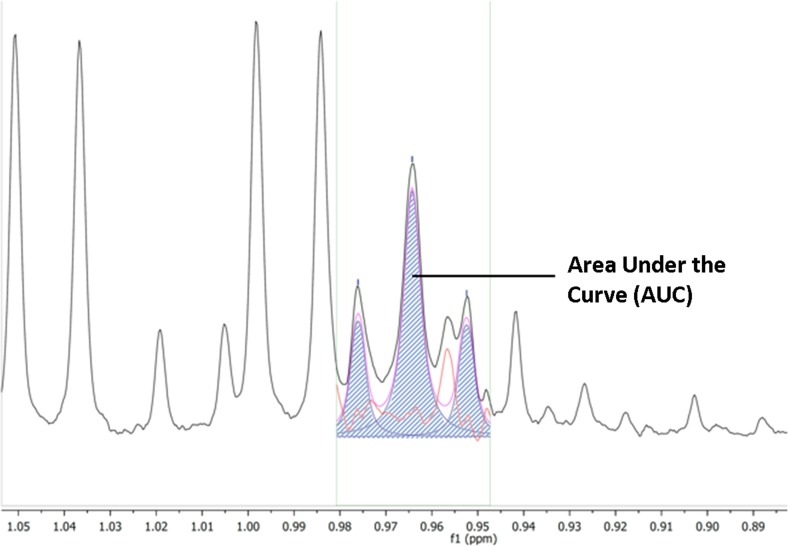
Quantification of the metabolites by area under the curve (AUC) The most resolved resonance was selected and fitted, noisy variables and spectral overlap eliminated before integration (MestReNova software). AUC (dashed area) is expressed in arbitrary units.

The VIP ≥ 1 metabolites were then identified using in-house references, ChenomX (Version 8.1, ChenomX Inc., Canada), the Human Metabolome DataBase and published data (Figure 5) [71, 4]. Most of the identified metabolites were level 2, according to the Metabolomics Standard Initiative (MSI) classification [72]. For quantification of each discriminant metabolite, the most resolved resonance was selected and fitted. Noisy variables and spectral overlap were eliminated before integration (MestReNova software). An area under curve (AUC) in arbitrary units was obtained. It allowed statistical comparison between sub-groups of patients [73] (Figure 6).

### Statistical analysis

In order to compare the clinical characteristics among patients clusters, we used Welch two-samples *t*-tests for MBC data (age, BMI and overall survival). For EBC data, we used ANOVA for age and Body Mass Index (BMI) associated with Pairwise Multiple Comparison Procedure (Tukey Test), Kruskal-Wallis on Ranks for tumor size and Fischer test for tumor intrinsic subtypes and tumor staging (TNM).

For comparison of median metabolites values between patients clusters, we used Wilcoxon test for MBC clusters and Kruskal-Wallis one Way Analysis of Variance on Ranks for EBC clusters and whole population associated with significant Pairwise Multiple Comparison Procedures (Dunn's Method) if significant. False Discovery Rate correction was applied using Bonferroni method. Adapted p values are shown in the corresponding legends [74].

Pearson correlation test was used for correlation between metabolites AUC values and tumor size, BMI and for correlations between metabolites levels themselves associated in this case with Pairwise two-side test if significant.
